# Coding and description of growth stages of precocious walnut in Xinjiang based on the extended BBCH scale

**DOI:** 10.3389/fpls.2026.1863766

**Published:** 2026-07-07

**Authors:** Jingyu Zhao, Zhongzhong Guo, Yang Wang, Shan Gao, Shangqi Yu, Yongqiang Chen, Qiang Jin, Rui Zhang

**Affiliations:** 1 College of Horticulture and Forestry, Tarim University, Alar, China; 2 Southern Xinjiang Distinctive Foresty and Pomology Technology Innovation Center, Xinjiang Production and Construction Corps, Alar, China; 3 Huazhong Agricultural University - Tarim University Southern Xinjiang Horticulture Research Center, Alar, China; 4 College of Life Science and Technology, Tarim University, Alar, China; 5 The National-Local Joint Engineering Laboratory of High Efficiency and Superior-Quality Cultivation and Fruit Deep Processing Technology on Characteristic Fruit Trees, Tarim University, Alar, China

**Keywords:** germplasm resources, *Juglans regia*, orchard management, phenology, reproductive development, Xinjiang

## Abstract

**Introduction:**

Xinjiang is one of the important origins of walnut (*Juglans regia* L.) and harbors abundant germplasm resources. However, a standardized phenological description system for precocious walnuts in this region is lacking. This study aimed to establish such a system based on the extended BBCH scale.

**Methods:**

We conducted systematic phenological observations over two growing seasons (2023 -2024) on six nine-year-old precocious walnut materials (two main cultivars and four elite strains) planted in Alar, Xinjiang. The study covered eight principal growth stages: bud development (0), leaf development (1), shoot development (3), inflorescence emergence (5), flowering (6), fruit development (7), fruit maturity (8), and senescence and dormancy (9), further subdivided into 38 secondary stages, with coded morphological photographs provided for each stage.

**Results:**

Significant differences were observed among varieties and elite strains. BT-2 had the earliest budburst, the highest number of female flowers, and reached standard fruit size first; XW-185 had abundant male flowers and could serve as a suitable pollen donor; BT-1 exhibited the highest kernel percentage and the thinnest nutshell. Correlation analysis revealed that the trait correlation patterns of BT-2 and BT-3 were relatively complex, showing opposite trends. PCA and clustering heatmap indicated clear differentiation among the six materials, classifying them into four clusters.

**Discussion:**

The BBCH_based system provides a practical tool for pollinizer arrangement, irrigation scheduling, and breeding of precocious walnuts. Complementary traits among varieties support targeted utilization, and phenological stages can guide stage_specific irrigation. However, the two_year observation period limits the assessment of long_term stability, and future research should further elucidate the interaction network between early fruiting traits and quality traits.

## 1 Introduction

Walnut (*Juglans regia* L.), which originated in ancient Persia, is now widely cultivated in temperate and semi-arid regions of Asia, Europe, and America (Vahdati et al., 2019). This extensive cultivation results from its environmental adaptability and its rich nutritional profile. Walnuts are high in unsaturated fatty acids (Li P. et al., 2024), polyphenols (Wu S. T. et al., 2021), flavonoids (Aneklaphakij et al., 2021), and essential amino acids (Wu et al., 2020). Furthermore, polyunsaturated fatty acids (Kafkas et al., 2017; Goodarzi et al., 2023) and minerals (Trandafir et al., 2016) also contribute positively to human health. Within China, there exists a significant diversity of wild and cultivated walnut resources. Notably, the Xinjiang region is home to a type of precocious walnut. The seedlings of this variety can differentiate female flower buds 1 to 3 years after sowing, rendering them valuable for the study of precocious flowering. To systematically describe plant growth and development stages, introduced a universal scale that employs decimal codes to characterize the growth stages of various crops, known as the BBCH scale (Lancashire et al., 1991).Research on the BBCH scale was initially not applied to the developmental stages of fruit trees. However, following extensive studies on the BBCH scale in crops, it has become widely utilized across various fruit trees species, including mulberry (Mo et al., 2022), pear (Martínez et al., 2019), kiwi (Salinero et al., 2009), papaya (Ferrer-Blanco et al., 2022), chestnut (Larue et al., 2021), mango (Hernández Delgado et al., 2011), cashew tree (Adiga et al., 2019), *Xanthoceras chinensis* (Jiang et al., 2024), persimmon (Guan et al., 2021), and longan (Pham et al., 2015). Currently, systematic phenological descriptions for precocious walnuts are still inadequate, particularly lacking detailed stage division and visual documentation based on a standardized phenological scale. Accurate and standardized phenological stage descriptions are essential for subsequent research, genetic improvement, and field management of walnut. Mastering such detailed phenological information is of great significance for scientifically guiding agronomic practices such as fertilization, irrigation, and pruning. This study adopted the BBCH scale to comprehensively and systematically describe the phenological stages of walnut. For each BBCH stage, corresponding morphological photographs were provided to facilitate visual identification of phenological changes in precocious walnuts. In addition, related traits including leaf area, flower buds, green fruits, and dried fruits were measured and subjected to correlation analysis to provide a reference for new variety breeding. Standardized phenology can systematically guide orchard pollinizer arrangement, water and fertilizer management, and cross-breeding programs, while providing a practical technical basis for adaptability evaluation and standardized production of precocious walnuts in this region.

## 2 Materials and methods

### 2.1 Experimental area

**Figure 1:**
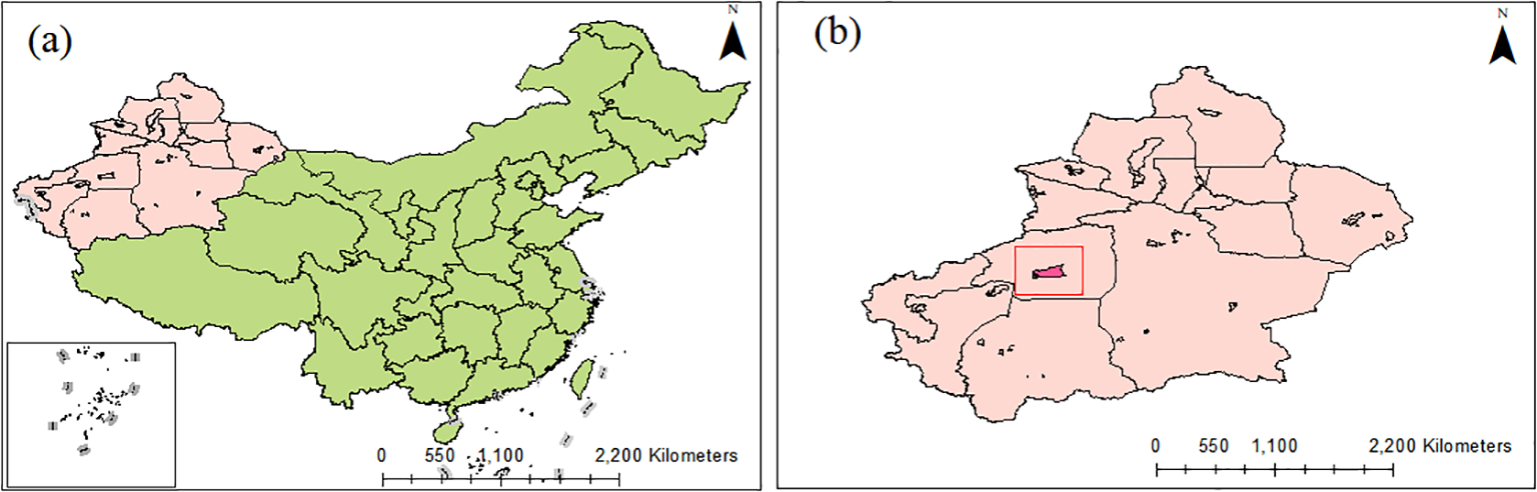
Experimental area map. **(a)** Map of China, **(b)** map of Alar City, Xinjiang.

The experimental site is located in the Southern Xinjiang Walnut Germplasm Repository in Alar City, Xinjiang, China (N40°23′, E80°03′), at the northwestern edge of the Taklimakan Desert (**Figure 1**). The site is at an average altitude of 1,049 m and features a warm temperate continental arid desert climate, with abundant light and heat resources and a large diurnal temperature range. The annual mean temperature is 11 °C, with a maximum of 43.9 °C and a minimum of -27.1 °C. The annual mean precipitation is 65 mm, the annual mean evaporation is 2,337.5 mm, and the annual sunshine duration is 2,793.4 h.

### 2.2 Plant materials and phenological evaluation

For this experiment, six walnut varieties and elite strains with a tree age of nine years, consistent growth, and free from diseases and pests were selected from trees that were already planted. The trees are planted in a north-south direction with a spacing of 3m×5m.Three sample trees of each variety were chosen and marked with tags. Varieties name of XW-185 and XX-2, elite strains name BT-1, BT-2, BT-3 and BT-4. Among them, ‘B’ stands for the Xinjiang Production and Construction Corps (derived from the initial letter of the Chinese pinyin ‘Bing’), ‘T’ stands for Tarim University (derived from the initial letter of the Chinese pinyin ‘Ta’), and the numbers 1 to 4 indicate the codes of the elite strains. For each tree, 30 fruiting branches are randomly selected from the tree crown for observation. The observation frequency is adjusted according to the growth and development rate of the walnuts. Given that from January to May, walnuts undergo multiple critical phenological stages with rapid morphological changes, such as bud development, leaf development, flowering and fruit enlargement stage, it is necessary to observe them every 2 to 3 days to precisely capture the dynamic process. After June, the fruit enters a relatively slow growth period of hard kernel and kernel development, and the external morphological changes tend to stabilize. Therefore, it is chosen to observe once a week until the fruit is ripe. From the postharvest bud dormancy to the end of leaf fall, it lasts for two growing seasons (2023-2024). During the research period, ensure the normal fertilization and irrigation of the tree.

### 2.3 BBCH scale

In this study, the BBCH grading criteria for walnuts were delineated using eight of the ten primary stages, which include bud development (0), leaf development (1), shoot development (3), inflorescence emergence (5), flowering (6), fruit development (7), fruit maturity (8), and senescence and beginning of the rest period (9) (Robin et al., 2024). The principal growth stages 2 (formation of lateral branches/tillers) and 4 (development of harvestable vegetative plant parts or vegetative reproductive panicle formation) were excluded from consideration. Additionally, the secondary growth stage is encoded with values ranging from 0 to 9, reflecting the growth sequence or percentile value. For example, a value of 5 for the main growth stage 1 (leaf development) indicates that leaf spread has reached approximately 50% of the entire tree, thereby defining this stage as 15. Similarly, a value of 7 for the main growth stage 7 (fruit development) corresponds to 77, signifying that the fruit has attained 70% of its final size.

### 2.4 Statistical analysis

During the dormancy period, the total number of flower buds, the number of female flowers, the number of male inflorescences, the length of male inflorescences, and the number of male flowers per male inflorescence were recorded on the fruiting shoots of each tree. The ratio of female flowers to male inflorescences was also calculated. For each variety and elite strain, 30 male inflorescences were selected; their length was measured using a vernier caliper (0–200 mm, Guilin, China), and the number of male flowers per inflorescence was recorded. On each fruiting shoot, 30 leaves were randomly selected and photographed using the grid method, and leaf area was calculated using Image software. Starting from five days after flowering, the transverse diameter, longitudinal diameter, and lateral diameter of green fruits of the six precocious walnut varieties and elite strains were recorded every five days until 80 days after flowering. During fruit development, three walnuts from each variety and elite strain were harvested, cut transversely, and stained with 3% phloroglucinol solution for lignin observation, followed by photography. After fruit maturity, 30 fruits were collected from each variety and elite strain. The single fruit weight and kernel weight of dried fruits were measured using an electronic analytical balance (METTLER TOLEDO PL403, 410 g max, 0.001 g resolution), and the kernel percentage was calculated. The nutshell thickness was measured using a vernier caliper. Image organization and analysis were performed using Adobe Photoshop 2025, and statistical analysis was conducted using GraphPad Prism 6.0 and Origin 2021 software. All results are presented as means, and differences were considered significant at *P* < 0.05.

## 3 Results

### 3.1 Phenological period coding for walnuts

The extended BBCH scale categorizes the plant growth cycle into ten primary stages, numbered 0 to 9. This study provides a comprehensive description of eight of these stages, which encompass bud development (0) to nearly complete leaf shedding (9). Additionally, a total of 38 distinct secondary growth stages were documented (**Table 1**). Herein, ‘S’ represents the male inflorescence, and ‘P’ represents the female flower.

**Table 1:** BBCH codes and phenological periods for six precocious walnut varieties and elite strains.

BBCH code	Description
Principal growth stage 0: Bud development (Figure 2.)	
00	Dormancy stage: Characterized by closed leaf and flower buds covered with brown protective scales.
03	Bud swelling completion: Brown bud scales begin to separate slightly, indicating termination of dormancy phase.
07	Bud break initiation: Leaf tips become visibly emergent through separating bud scales, marking the onset of primary foliar development.
09	Green tip stage: Distinct green leaf tips visibly extend 5–10 mm beyond the bud scales, confirming active shoot elongation.
Principal growth stage 1: Leaf development (Figure 3.)	
10	Separation of the first leaf blade: The green scales open slightly.
13	More leaves unfold: The petiole can be seen.
15	More leaves unfold: Approximately accounting for 50% of the final unfolded state.
17	More leaves unfold, approximately accounting for 70% of the final unfolded state.
19	All leaves are fully unfolded: The size of the leaves no longer changes.
Principal growth stage 3: Shoot development (Figure 4.)	
31	New shoot begins to grow: The new shoot is light green, and the branch is approximately 10% of the final length.
33	The new shoot continues to grow: The branch is approximately 30% of the final length.
35	The new shoot continues to grow: The branch is approximately 50% of the final length.
37	The new shoot continues to grow: The length of the new shoot accounts for approximately 70% of the total length.
39	The new shoot continues to grow: The branch is approximately 90% of the final length.
Principal growth stage 5: Inflorescence emergence (Figure 5.)	
51S/51P	Male inflorescence bud: In a dormant state (stamens only), fully enclosed, covered with brown scales, and pointed at the tip. Female flower bud: The petals of female flowers appear.
53S/53P	Inflorescence bud swelling: The bud is closed and covered with light green scales. Female flower bud: The petals of female flowers are closed, which is 30% of the final size.
55S/55P	Inflorescence enlargement: The inflorescence starts to expand and elongate. Female flower bud: The petals of female flowers become larger, which is 50% of the final size.
57S/57P	Inflorescence elongation: The inflorescence is approximately 70% of the final length. Female flower bud: The petals of female flowers continue to grow larger, which is 70% of the final size.
59S/59P	The inflorescence continues to elongate: The inflorescence is approximately 90% of the final length. Female flower bud: The petals of female flowers are on the verge of opening, which is 90% of the final size.
Principal growth stage 6: Flowering (Figures 6, 7.)	
61S/61P	Male inflorescence: The style starts to open. Female flower: The petals slightly open.
65S/65P	Male inflorescence: The style fully opens and elongates. Female flower: The petals start to open.
67S/67P	Male inflorescence: The style elongates to its maximum length and starts to release pollen. Female flower: The petals fully open.
69S/69P	Male inflorescence: Withers and falls off. Female flower: Withers and wilts.
Principal growth stage 7: Fruit development (Figure 8.)	
71	Fruit setting: The diameter of the fruit is about 10 mm.
73	The fruit reaches 30% of its final size. The nuts start to expand rapidly.
75	The fruit is about 50% of its final size, and the hardening of the outer shell begins.
77	The fruit is about 70% of its final size, and the outer shell undergoes lignification.
79	The fruit stops growing: The fruit grows to the size of the standard variety.
Principal growth stage 8: Fruit maturity (Figure 9.)	
81	The internal structure of the fruit is preliminarily formed.
83	The internal wooden shell of the fruit begins to form, and the kernel is in a liquid state.
85	The fruit shell is preliminarily formed, and the kernel is in a gelatinous state.
87	The fruit shell is formed, and the kernel is plump.
89	The fruit is ripe; the green rind of the fruit begins to crack, and the fruit is fully mature.
Principal growth stage 9: Senescence and beginning of the rest period (Figure 10.)	
91	The growth of the branch is completed; the leaves are still completely green.
93	The leaves start to fade. Some leaves remain green while some turn yellow.
95	The beginning of leaf fall: The leaves start to drop, and the number of yellow leaves increases.
97	More leaves drop: 70% of the leaves have dropped.
99	The end of leaf fall: Almost all the leaves have fallen.

### 3.2 Comparison of phenological periods among walnut varieties and elite strains

**Figure 2:**
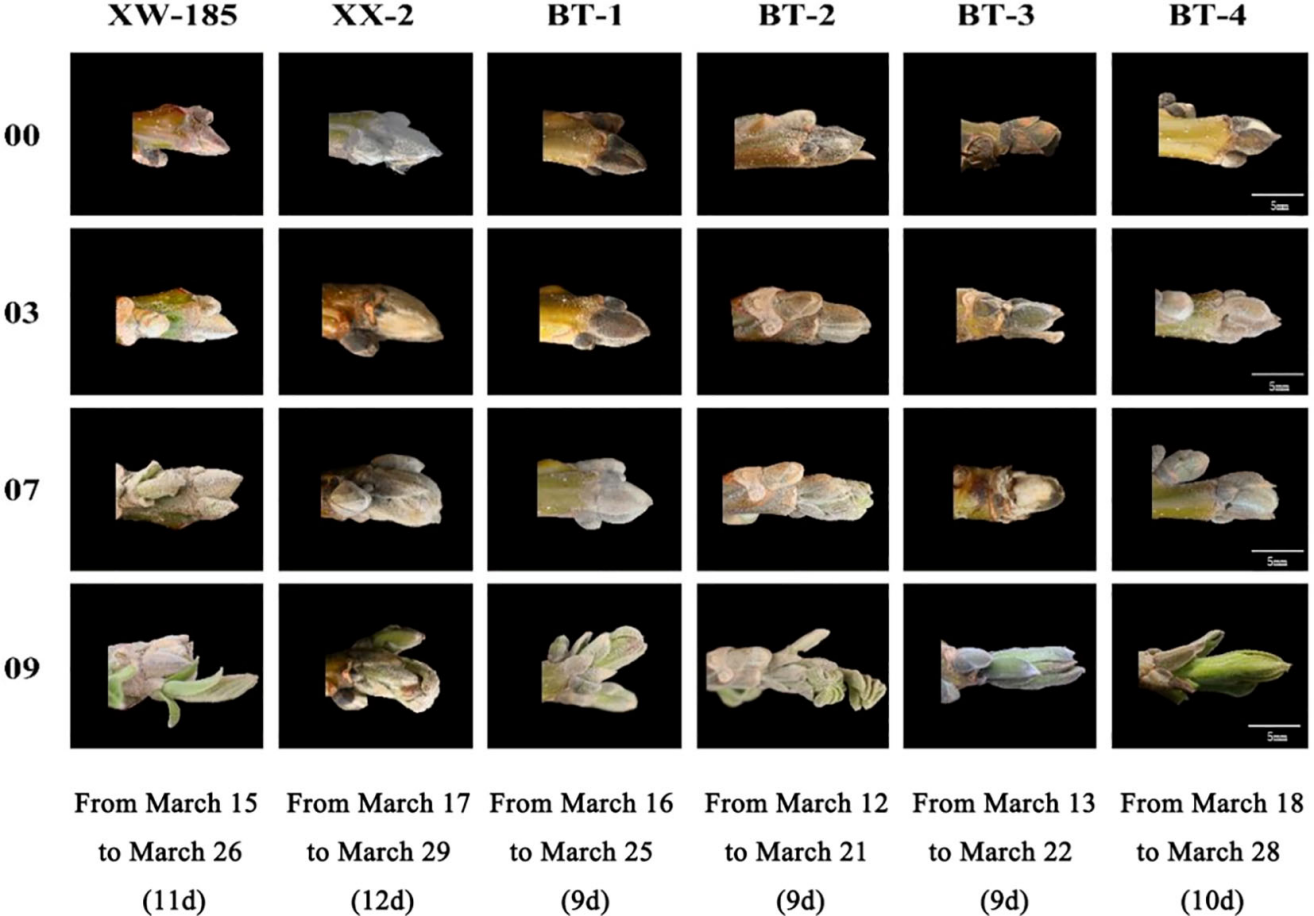
BBCH stage 0: bud development.

**Figure 3:**
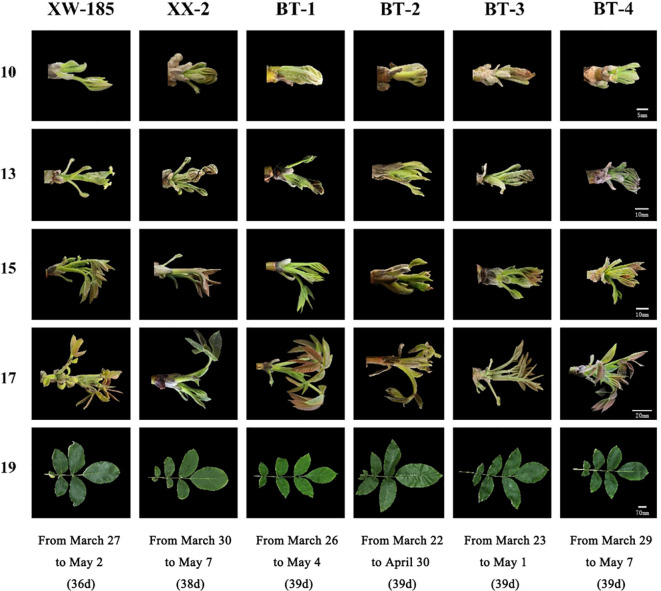
BBCH stage 1: leaf development.

**Figure 4:**
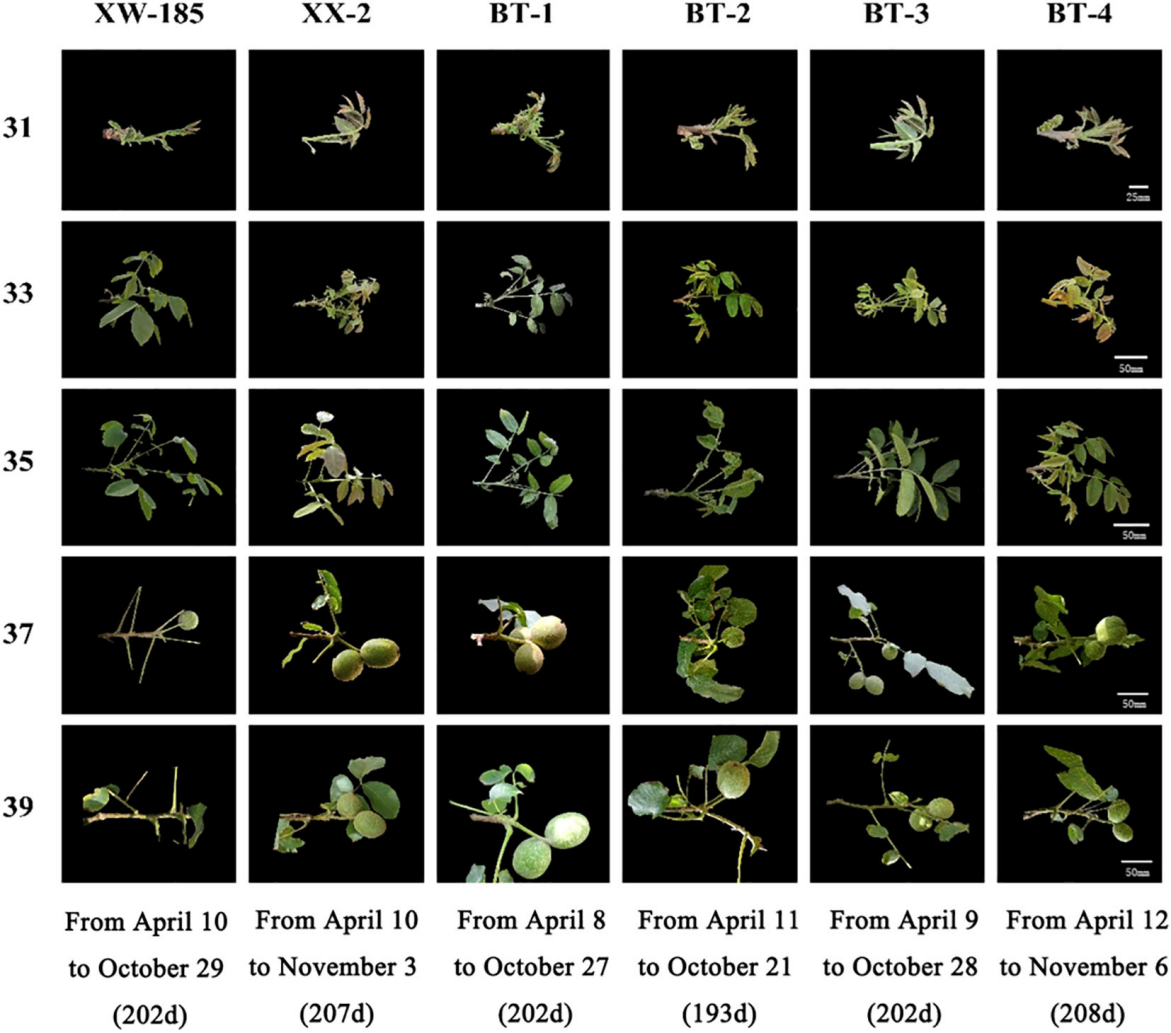
BBCH stage 3: shoot development.

**Figure 5:**
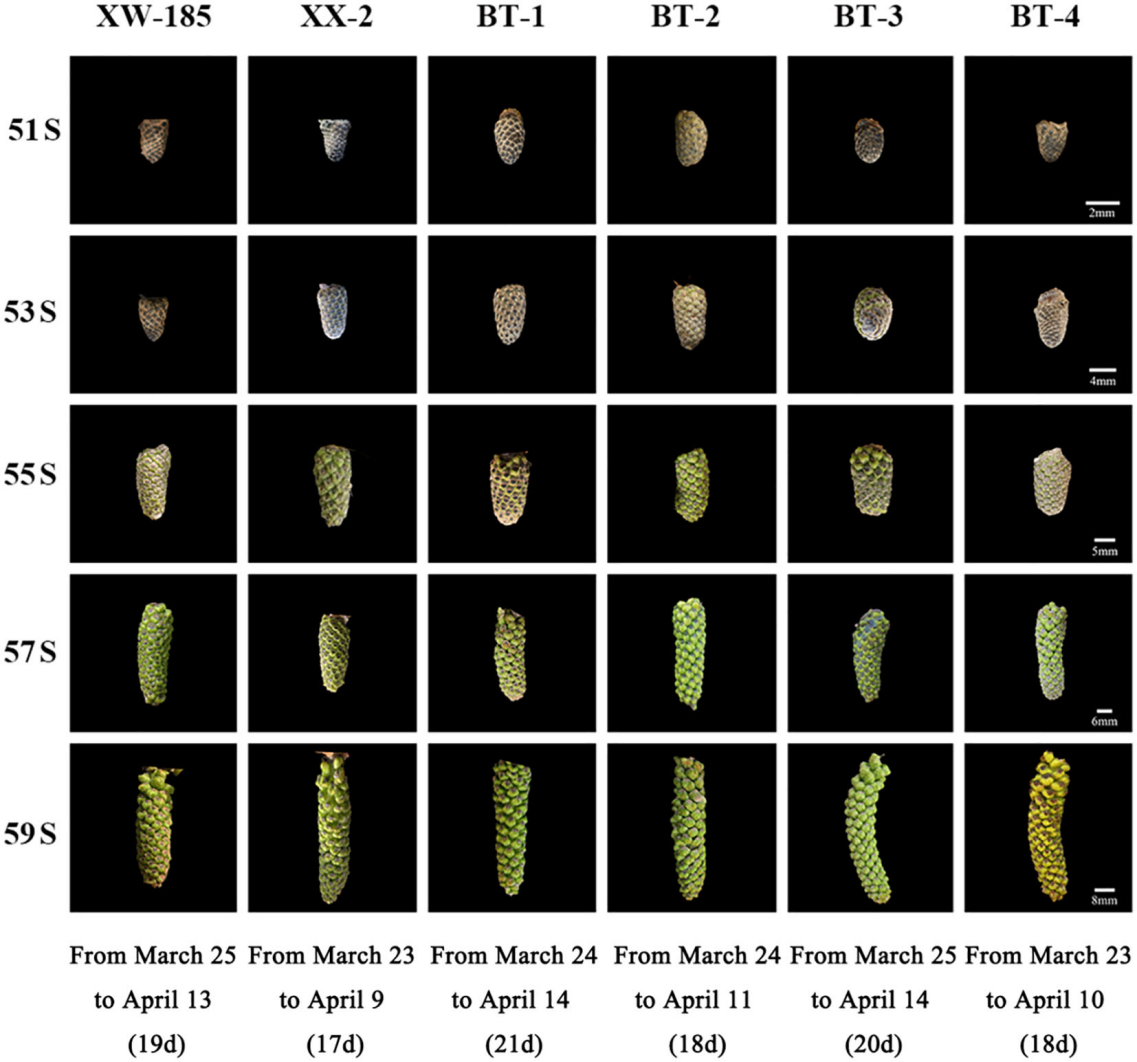
BBCH stage 5: inflorescence emergence.

**Figure 6:**
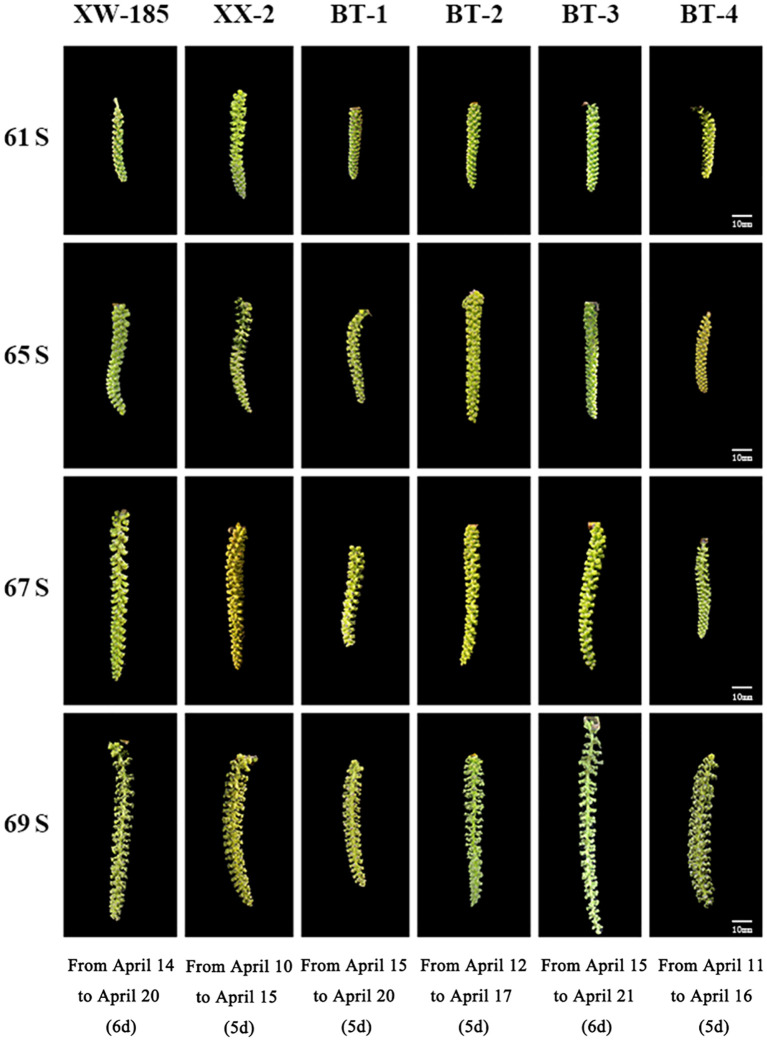
BBCH stage 6: male inflorescence development.

**Figure 7:**
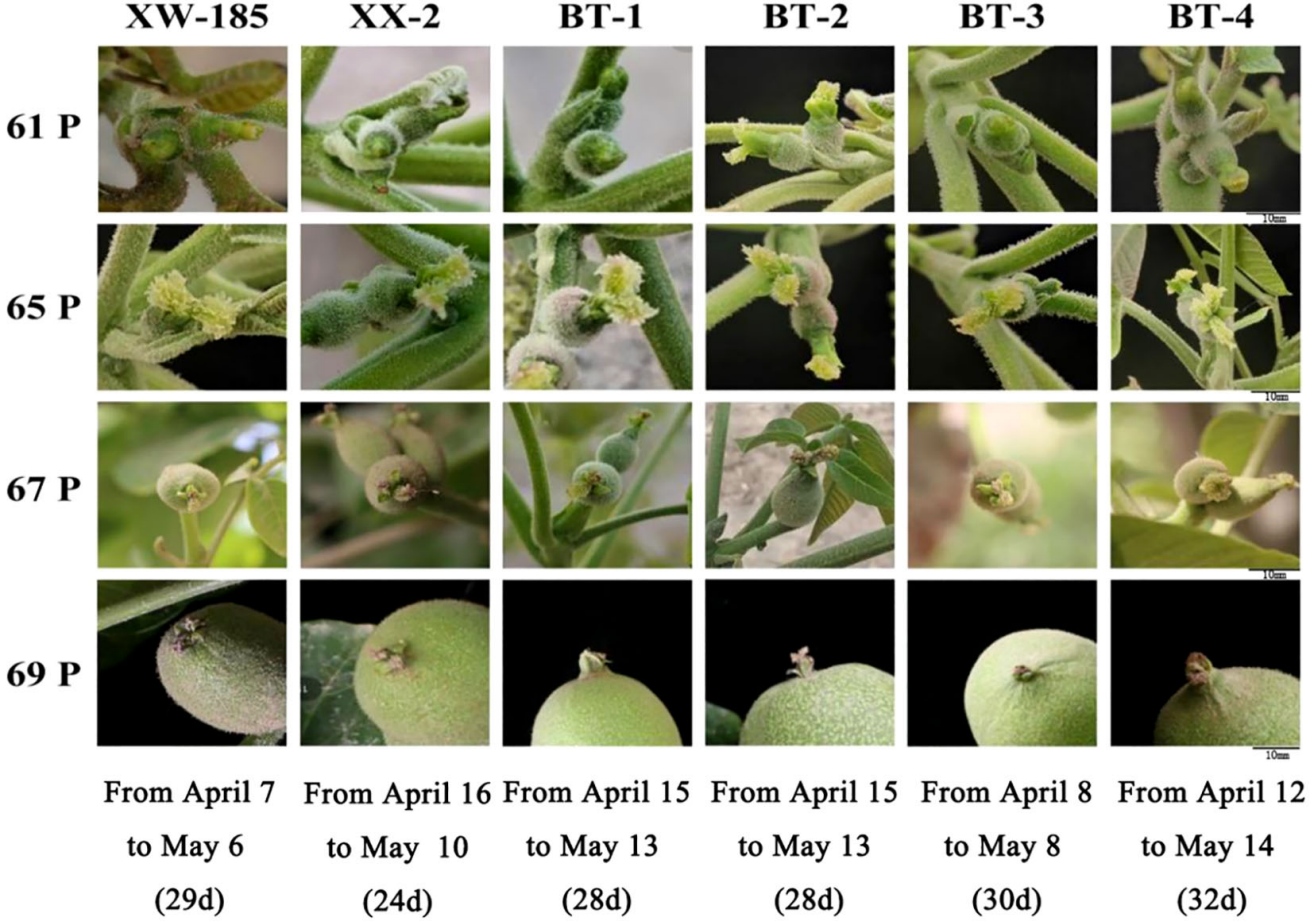
BBCH stage 6: female flower development.

**Figure 8:**
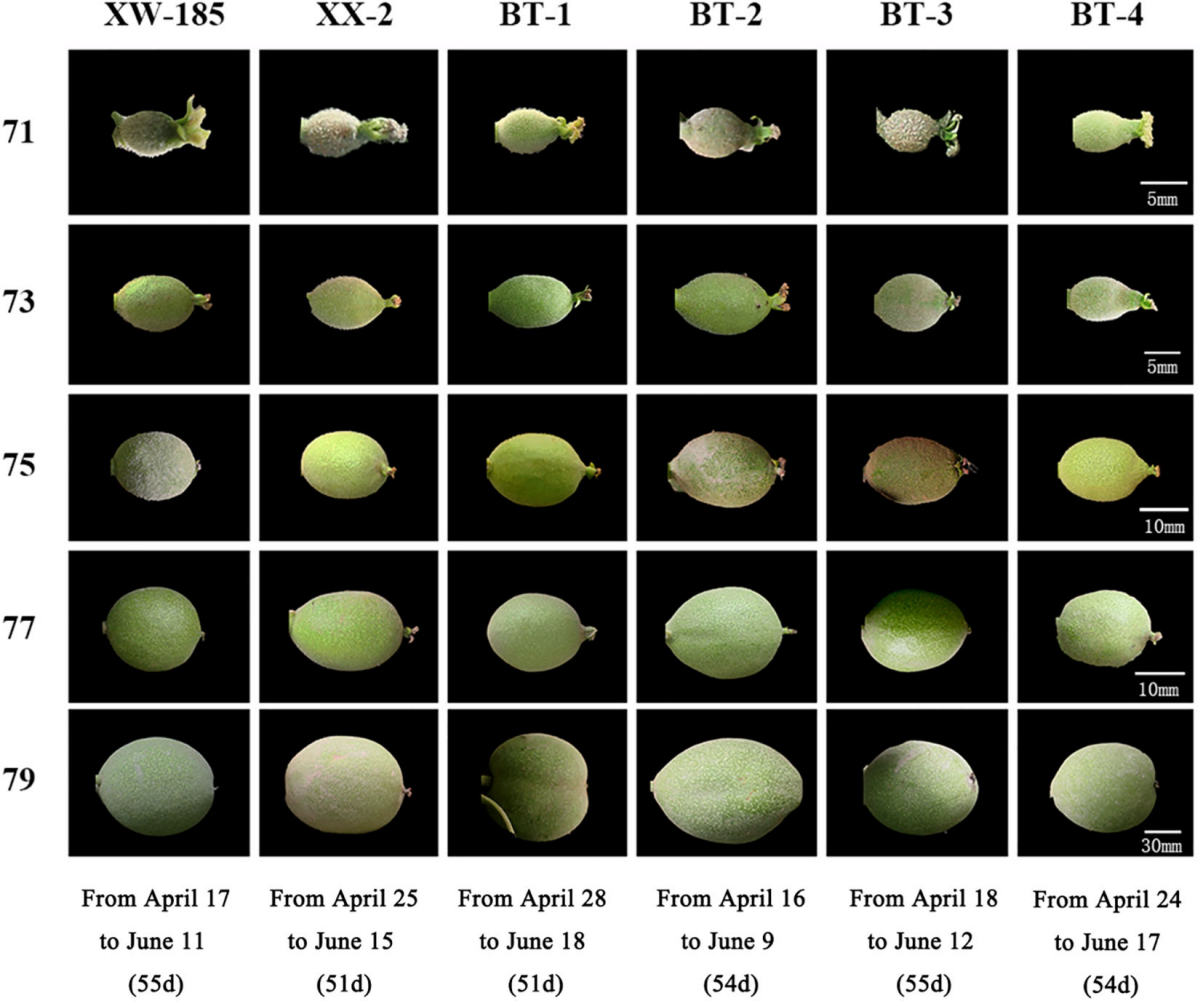
BBCH stage 7: fruit development.

**Figure 9:**
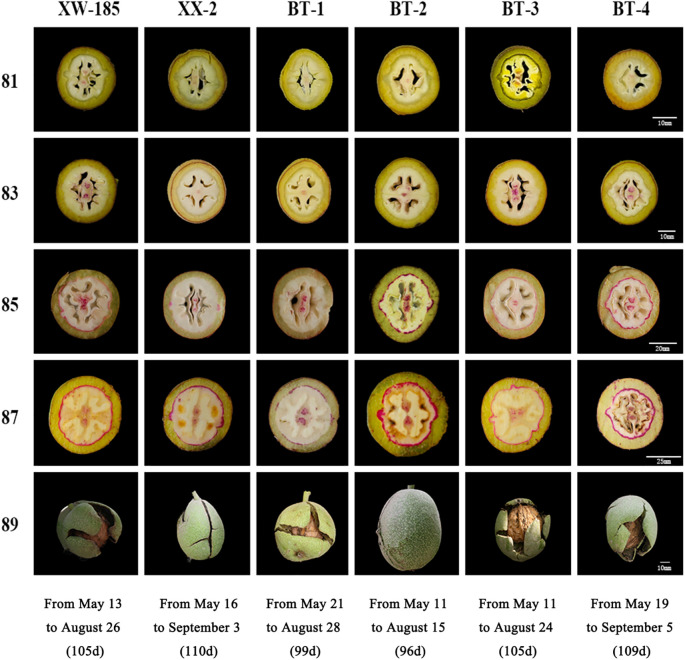
BBCH stage 8: fruit maturity.

**Figure 10:**
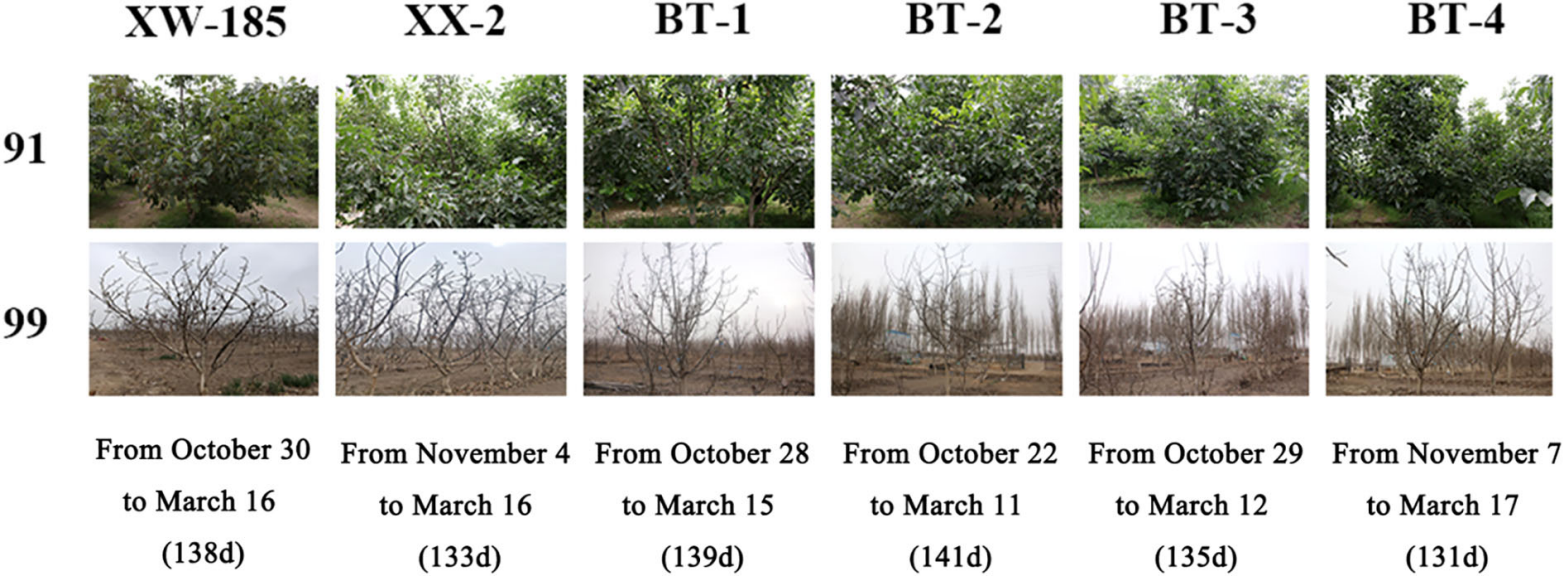
BBCH stage 9: senescence and beginning of the rest period.

**Figure 11:**
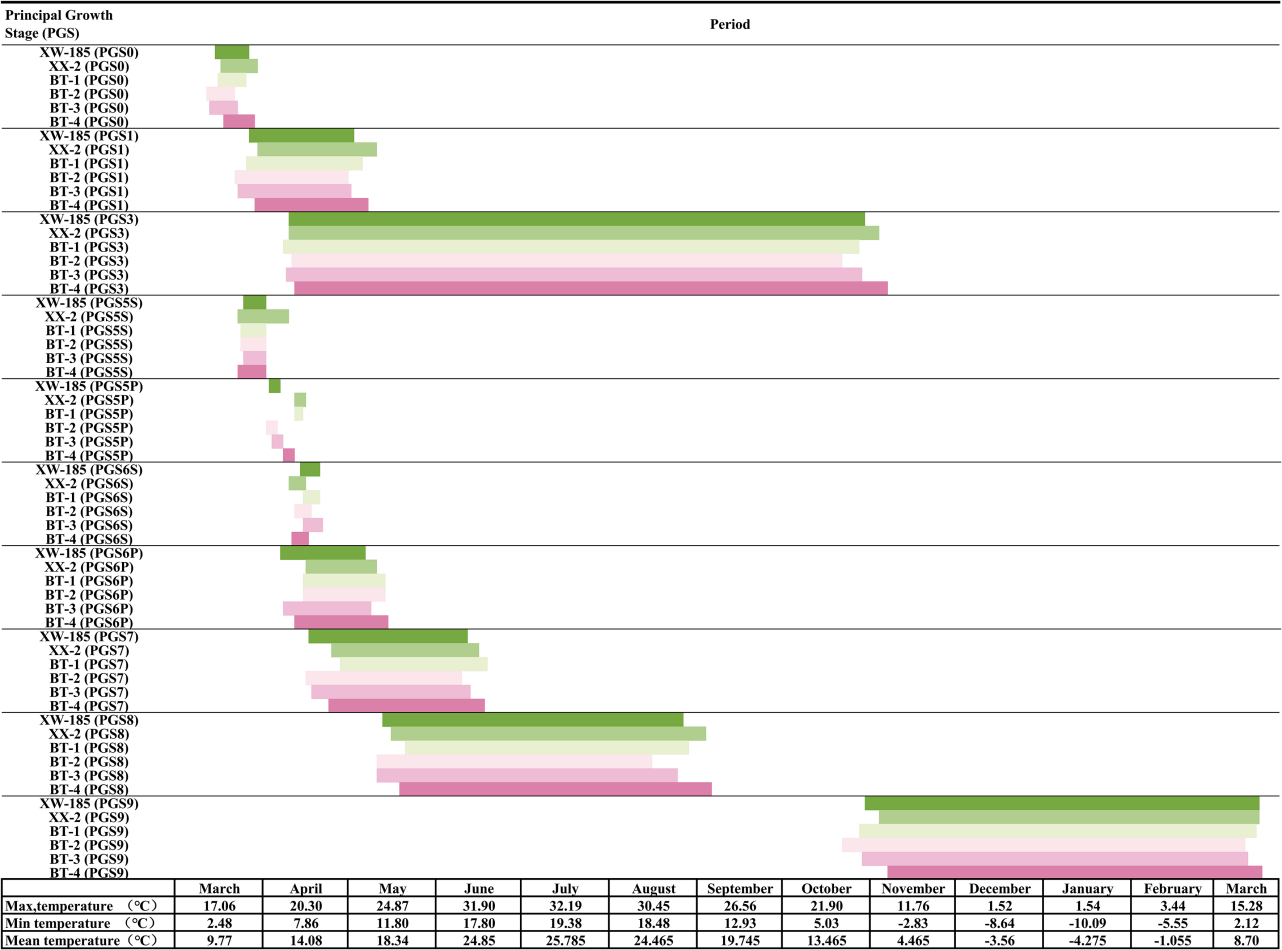
Schematic diagrams of growth stages for six precocious walnut varieties and elite strains (2023–2024).

To illustrate the phenological processes of different precocious walnut varieties and elite strains, the corresponding photographs were arranged according to the BBCH scale codes, forming a matrix with principal growth stages as rows and different varieties and elite strains as columns, with the duration of each stage indicated below the photographs, as shown in **Figures 2**–**10**. **Table 2** presents the comparative analysis of flower bud indicators. Furthermore, based on the phenological stages, BBCH codes, and corresponding monthly mean temperatures of each variety and elite strain, a growth and development Gantt chart was constructed, as shown in **Figure 11**.

**Table 2:** Analysis of differences in flower bud indicators among six precocious walnut cultivars and elite strains.

Variety	Number of flower buds (No.)	Female flower number (No.)	Male inflorescence number (No.)	Female flowers/male inflorescences	Male inflorescence length (mm)	Male flower number (No.)
XW-185	9.33 ± 0.58 ab	3.67 ± 0.58 d	5.67 ± 0.58 a	0.66 ± 0.15 b	148.04 ± 9.61 a	118.56 ± 4.30 a
XX-2	8.67 ± 1.15 ab	5.67 ± 0.58 bc	3.00 ± 1.00 b	2.06 ± 0.82 ab	114.78 ± 2.39 b	96.56 ± 3.71 d
BT-1	9.00 ± 1.00 ab	6.00 ± 1.00 b	3.00 ± 1.00 b	2.22 ± 1.11 a	97.55 ± 4.12 c	96.67 ± 1.80 d
BT-2	10.67 ± 1.15 a	7.33 ± 0.58 a	3.33 ± 0.58 b	2.22 ± 0.19 a	102.01 ± 2.02 c	108.33 ± 1.50 c
BT-3	8.33 ± 0.58 b	4.67 ± 0.58 cd	3.67 ± 1.15 ab	1.38 ± 0.5 ab	147.26 ± 11.56 a	113.89 ± 2.42 b
BT-4	9.67 ± 1.53 ab	5.33 ± 1.15 bc	4.33 ± 2.08 ab	1.62 ± 1.22 ab	100.47 ± 2.18 c	95.56 ± 2.70 d

Data are presented as mean ± standard deviation. Lowercase letters indicate statistically significant differences at *p* < 0.05 among cultivars and elite strains.

**Figure 12:**
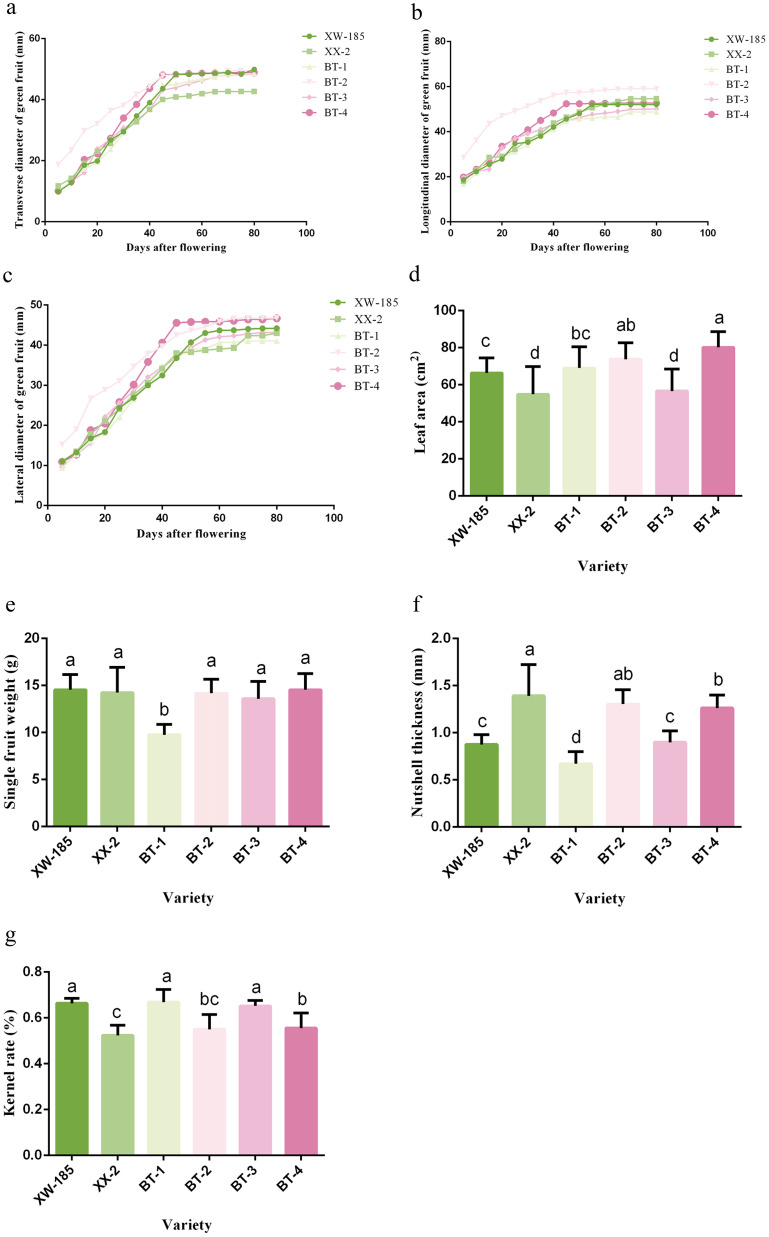
Analysis of leaf area and fruit external quality of six precocious walnut cultivars and elite strains. **(a)** Transverse diameter of green fruit, **(b)** longitudinal diameter of green fruit, **(c)** lateral diameter of green fruit, **(d)** leaf area, **(e)** single fruit weight of dried fruit, **(f)** nutshell thickness of dried fruit, **(g)** kernel rate of dried fruit.

Based on **Figures 2**-**10**, during the bud development stage (0), BT-2 had the earliest budburst, starting on March 12. Budburst in XX-2 and BT-4 occurred slightly later, on March 17 and March 18, respectively. The duration of bud development was nine days for BT-1, BT-2, and BT-3, while XX-2 had the longest bud development duration of 12 days. During the leaf development stage (1), BT-2 reached leaf expansion first on March 22, whereas XX-2 was the latest, on March 30. XW-185 had the shortest leaf development duration of 36 days. According to the leaf area data in **Figures 12D**, BT-4 had the largest leaf area (80.25 cm²), while XX-2 and BT-3 had the smallest leaf areas (54.88 cm² and 56.65 cm², respectively). During the shoot development stage (3), BT-1 showed the earliest shoot development on April 8, and BT-4 was the latest on April 12, four days later than BT-1. The shoot development duration of XW-185, BT-1, and BT-3 was 202 days each.

During the inflorescence emergence stage (5), the earliest male inflorescence emergence occurred in XX-2 and BT-4, both starting on March 23, while the latest was in XW-185 and BT-3, both on March 25. BT-1 had the longest male inflorescence emergence duration of 21 days. The earliest female flower emergence was observed in BT-2 and XW-185, on April 2 and April 3, respectively, and the latest was in XX-2 and BT-1, both on April 12. The duration of female flower emergence was approximately 2–3 days. During the flowering stage (6), the earliest male flowering occurred in XX-2 and BT-4, on April 10 and April 11, respectively, only one day apart. The earliest female flowering was in XW-185 on April 7, followed by BT-3 on April 8, and the latest were BT-1 and BT-2, both on April 15. According to **Table 2**, BT-2 had the highest total number of flower buds, while BT-3 and XX-2 had the lowest. BT-2 also had the highest number of female flowers, and XW-185 the lowest. XW-185 had the highest number of male inflorescences, while XX-2 and BT-1 had the lowest. The highest ratio of female flowers to male inflorescences was found in BT-1 and BT-2, both at 2.22. The longest male inflorescences were observed in XW-185 and BT-3 (148.04 mm and 147.26 mm, respectively), and the shortest in BT-1 (97.55 mm). The highest number of male flowers per male inflorescence was in XW-185 and BT-3 (118.56 and 113.89, respectively), and the lowest in BT-4 (95.56).

During the fruit development stage (7), precocious walnuts required 51–55 days to reach standard fruit size. BT-2 was the first elite strain to reach standard fruit size, on June 9, followed by XW-185 on June 11, while BT-1 was the latest, on June 18. During the fruit maturity stage (8), XX-2 and BT-4 had the longest fruit maturity durations of 110 and 109 days, respectively, while BT-2 and BT-1 had the shortest, at 96 and 99 days, respectively. During the senescence and dormancy stage (9), the earliest entry into senescence and dormancy was BT-2, on October 22, with a duration of 141 days, the longest dormancy period. The latest entry was BT-4, on November 7, 17 days later than BT-2, and BT-4 had the shortest senescence and dormancy duration of 131 days. **Figure 11** shows the growth stage schematic diagram of the six precocious walnut varieties and elite strains, including the timing of each stage as well as the monthly maximum, minimum, and mean temperatures.

### 3.3 Analysis of external fruit quality in walnut varieties and elite strains

To accurately compare the morphological characteristics of different precocious walnut varieties and elite strains, this study systematically measured the dynamic indicators of transverse diameter, longitudinal diameter, and lateral diameter of green fruits during the fruit development stage (7) of six precocious walnut varieties and elite strains in 2023, and measured the single fruit weight and nutshell thickness of dried fruits and calculated the kernel percentage during the fruit maturity stage (8). The results are shown in **Figure 12**. From **Figure 12A**, it can be seen that the transverse diameter of XW-185 green fruits entered the slow growth period at 50 days after flowering, reaching 48.33 mm, while the longitudinal and lateral diameters entered the slow growth period at 55 days after flowering, reaching 52.05 mm and 43.03 mm, respectively. For XX-2, the transverse diameter entered the slow growth period at 65 days after flowering (42.63 mm), and the longitudinal and lateral diameters entered at 70 days after flowering (54.61 mm and 42.38 mm, respectively). For BT-1, the transverse and longitudinal diameters entered the slow growth period at 70 days after flowering (48.36 mm and 48.74 mm, respectively), while the lateral diameter entered at 60 days after flowering (40.66 mm). For BT-2, the transverse diameter entered the slow growth period at 45 days after flowering (47.94 mm), and the longitudinal and lateral diameters entered at 65 days after flowering (58.94 mm and 46.67 mm, respectively). For BT-3, the transverse and longitudinal diameters entered the slow growth period at 70 days after flowering (48.08 mm and 49.92 mm, respectively), while the lateral diameter entered at 60 days after flowering (42.08 mm). For BT-4, the transverse, longitudinal, and lateral diameters all entered the slow growth period at 45 days after flowering (48.08 mm, 52.43 mm, and 45.58 mm, respectively).

In **Figure 12E**, the dried fruit weight of BT-1 was significantly lower than that of the other varieties and elite strains, at 9.75 g, while the differences among XW-185, XX-2, BT-2, and BT-4 were not obvious, all at 14 g. In **Figures 12F**, BT-1 had the thinnest nutshell thickness of only 0.67 mm, while XX-2 had the thickest at 1.39 mm, and BT-4 also had a relatively thick nutshell at 1.26 mm. According to **Figure 12G**, the highest kernel percentages were found in BT-1, XW-185, and BT-3, at 0.6692, 0.6639, and 0.6523, respectively, while the lowest was in XX-2, at 0.5235.

### 3.4 Analysis of correlation patterns in walnut varieties and elite strains

**Figure 13:**
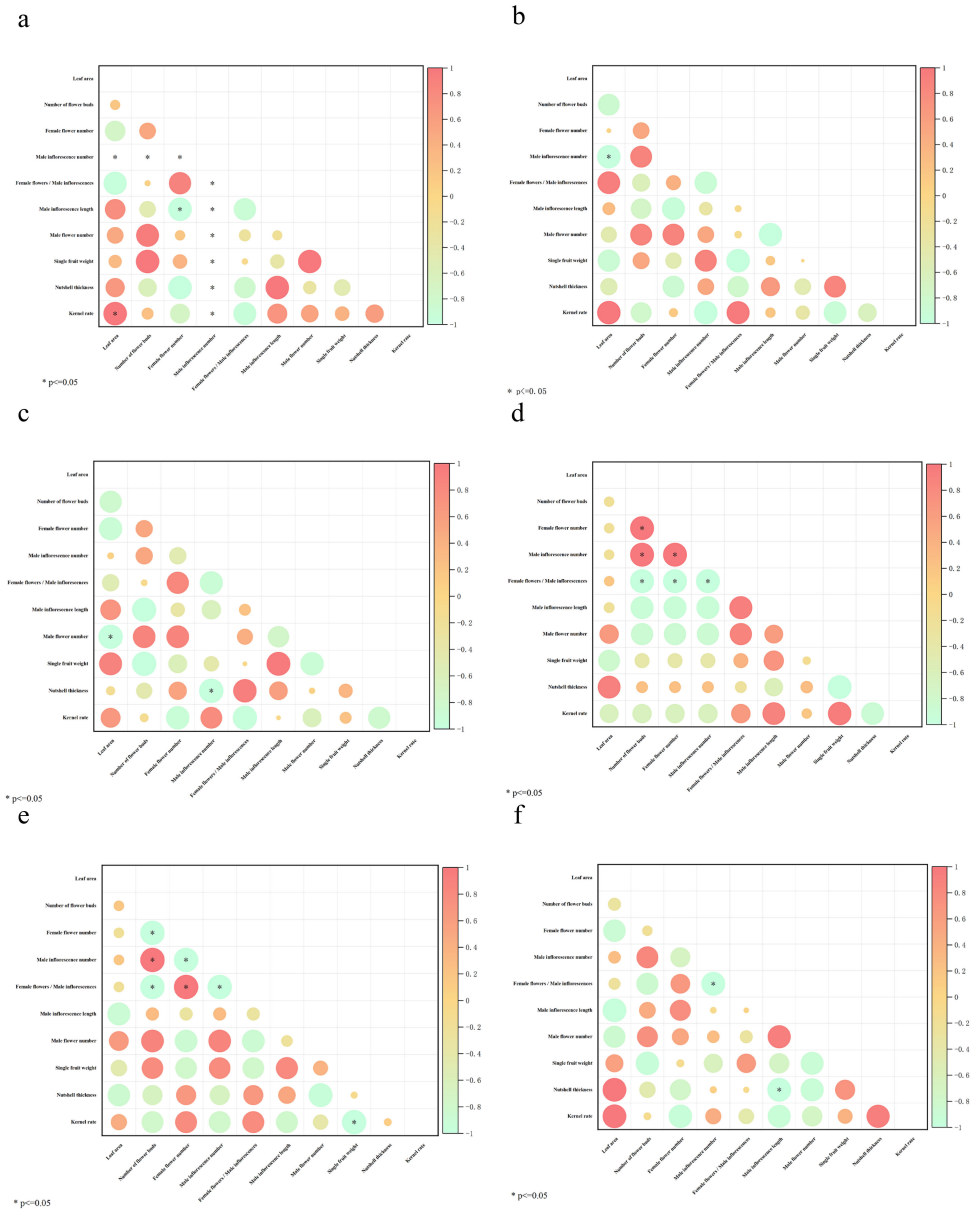
Trait correlation matrix of six precocious walnut varieties and elite strains. **(a)** Correlation analysis of XW-185, **(b)** correlation analysis of XX-2, **(c)** correlation analysis of BT-1, **(d)** correlation analysis of BT-2, **(e)** correlation analysis of BT-3, **(f)** correlation analysis of BT-4.

Correlation analysis of the related traits of six precocious walnut varieties and elite strains showed that the trait association patterns differed significantly among the materials (**Figure 13**). For XW-185, the overall correlations among traits were weak, with only leaf area showing a significant positive correlation with kernel percentage. For XX-2, leaf area was significantly negatively correlated with the number of male inflorescences, reflecting a competitive relationship between vegetative growth and male reproductive growth. For BT-1, the number of male inflorescences was significantly negatively correlated with nutshell thickness. For BT-2, the total number of flower buds was significantly positively correlated with both the number of female flowers and the number of male inflorescences, but significantly negatively correlated with the ratio of female flowers to male inflorescences. Furthermore, the number of female flowers was significantly positively correlated with the number of male inflorescences, and the ratio of female flowers to male inflorescences was significantly negatively correlated with both the number of female flowers and the number of male inflorescences. For BT-3, as the total number of flower buds increased, male inflorescences increased while female flowers decreased; the number of female flowers was significantly positively correlated with the ratio of female flowers to male inflorescences and significantly negatively correlated with the number of male inflorescences, indicating a male-biased resource allocation pattern. For BT-4, there were fewer significant correlations, manifested as a significant negative correlation between the number of male inflorescences and the ratio of female flowers to male inflorescences, and a significant negative correlation between male inflorescence length and nutshell thickness. Overall, the trait correlation patterns of BT-2 and BT-3 were relatively complex, with opposite trends in their association directions. XW-185 and XX-2 showed relatively independent trait associations. The synergistic or competitive relationships among traits such as flower buds, female flowers, male inflorescences, leaf area, and fruits were clearly differentiated among different precocious walnut materials, providing a basis for targeted cultivation management and variety improvement.

### 3.5 Principal component and cluster analysis of walnut varieties and elite strains

**Figure 14:**
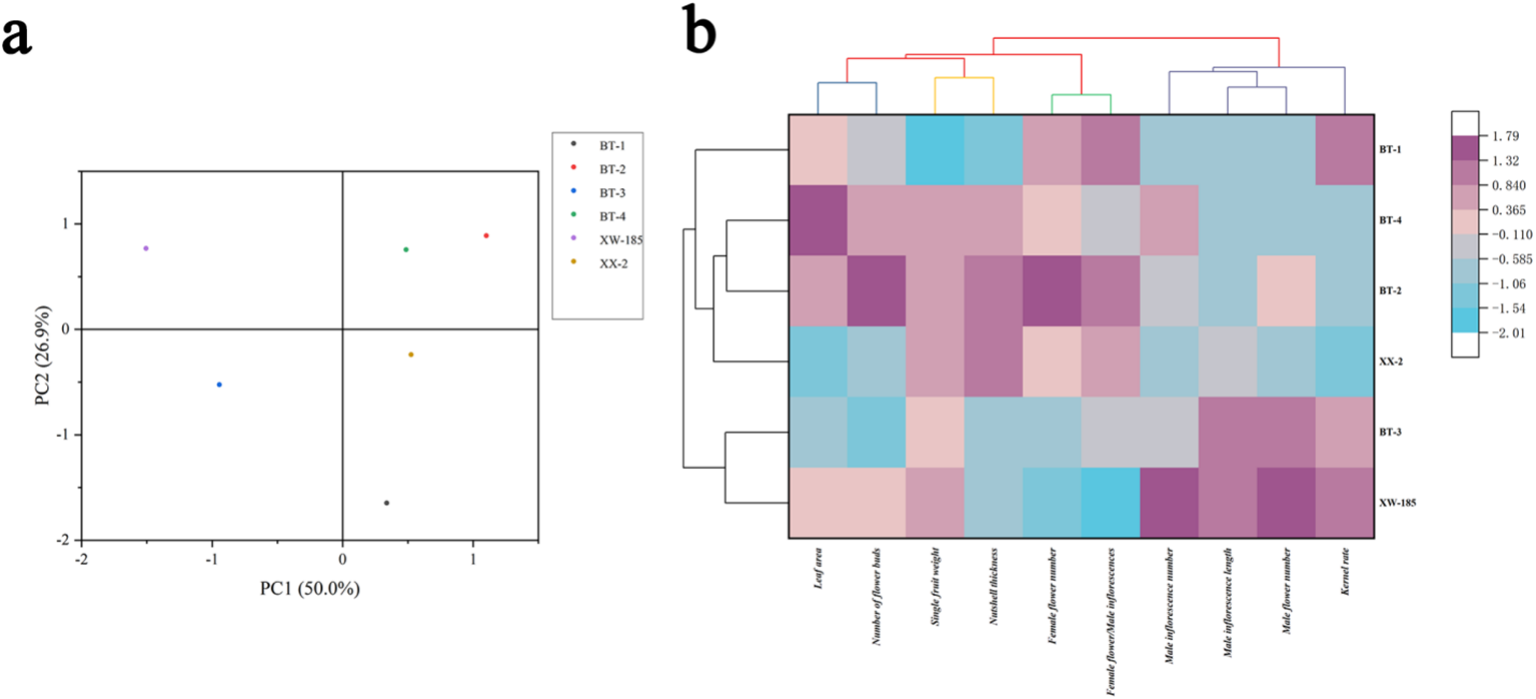
Principal component analysis and hierarchical clustering heatmap of six precocious walnut varieties and elite strains. **(a)** Principal component analysis (PCA) biplot, **(b)** hierarchical clustering heatmap.

Principal component analysis (PCA) biplot (**Figure 14A**) showed that the first two principal components explained 50.0% and 26.9% of the total variance, respectively, with a cumulative contribution of 76.9%, indicating that they captured most of the overall variation in the measured traits. The six precocious walnut varieties and elite strains were clearly separated from each other in the coordinate system, suggesting significant overall phenotypic differentiation among them. PC1 was primarily associated with male inflorescence length (loading -0.415) and the ratio of female flowers to male inflorescences (loading 0.406), while PC2 was mainly associated with single fruit weight (loading 0.530) and nutshell thickness (loading 0.404). The hierarchical clustering heatmap (**Figure 14B**) further refined the similarity relationships among the varieties. Based on Euclidean distance, the six materials were classified into four clusters: Cluster I contained only BT-1; Cluster II comprised BT-2 and BT-4; Cluster III consisted of XX-2 alone; and Cluster IV included BT-3 and XW-185. The color blocks in the heatmap visually revealed distinct expression patterns of traits such as leaf area, number of flower buds, female flower number, and male inflorescence length among the clusters. For example, Cluster II exhibited higher leaf area and female flower number, whereas Cluster IV had longer male inflorescences and more male flowers. In summary, the results of PCA and cluster heatmap support each other, demonstrating that the six varieties and elite strains possess stable and distinguishable differences in the relevant phenotypic traits, which are sufficient to differentiate the materials and provide data support for parental selection in cross-breeding.

## 4 Discussion

### 4.1 Phenological period division and analysis of basic flowering characteristics

Utilizing the extended BBCH scale, this study systematically characterized eight primary growth stages and 38 secondary growth stages of six precocious walnut varieties and elite strains in Xinjiang. A comprehensive Gantt chart was developed to illustrate the progression from bud awakening to dormancy. This coding system provides a standardized and quantifiable descriptive language for each developmental stage of walnuts. The initial systematic observation of all BBCH stages was performed on precocious walnut germplasm in Xinjiang, elucidating the critical phenological milestones for each variety under local climatic conditions. This research establishes a foundational timeline for subsequent cultivation management and variety selection.

In terms of flowering biology, walnut exhibits the characteristic of heterodichogamy, where the asynchronous flowering periods of male and female flowers can severely affect pollination and fruit set rates, thereby limiting yield and quality (Li H. X. et al., 2024). To avoid the ‘inbreeding depression’ associated with self-pollination, walnuts and other plants have evolved two types of asynchronous flowering (Groh et al., 2025). The male inflorescences of the six tested precocious walnut varieties and elite strains all developed earlier than the female flowers. Among them, XW-185 had the highest number of male flowers per male inflorescence but the lowest number of female flowers, while BT-2 had the highest number of female flowers with moderate male inflorescence length and male flower quantity, and XX-2 had a relatively high number of female flowers but shorter male inflorescences. Studies have shown that when female flowers are pollinated to enhance reproductive success, plants correspondingly increase the number of female flowers and reduce the proportion of male flowers (Gao et al., 2021). The above flowering characteristics form a clear complementary pattern: XW-185 can provide abundant male flower resources, while BT-2, as a female-flower-rich receptor, can achieve a relatively high cross-pollination fruit set rate.

### 4.2 Analysis of flowering characteristics and fruit appearance quality

Analysis of flowering data and fruit quality indicators indicates that BT-2 exhibits the highest number of female flowers and a relatively high total number of flower buds. Furthermore, it reaches the standard fruit size earliest during the fruit development stage, and its single fruit weight shows favorable performance. This finding suggests that abundant female flower resources and an earlier onset of fruit development may be critical prerequisites for this elite strain to achieve greater single fruit weight and earlier maturity. In contrast, although the single fruit weight of BT-1 is significantly lower, it possesses the highest kernel percentage, the thinnest nutshell thickness, and the greatest ratio of female to male flowers. This balanced sex allocation may facilitate the effective distribution of reproductive resources. Several studies have indicated that the new variety ‘Helete Güneşi’ demonstrates delayed leaf development and earlier maturation relative to its parent varieties, in addition to exhibiting a higher kernel percentage. These observations are consistent with the early maturity traits of BT-2 and the elevated kernel percentage of BT-1 noted in this study (Sütyemez et al., 2021). Previous evaluations of walnut germplasm have also indicated that nutshell thickness is significantly negatively correlated with kernel percentage, while in the present study, the single fruit weight of BT-3 was significantly negatively correlated with kernel percentage (Khadivi et al., 2019).

The walnut shell is the endocarp, which is primarily composed of cells with thickened cell walls (Hu et al., 2018). The plant cell wall is a complex structure mainly consisting of high-molecular-weight polysaccharides, highly glycosylated proteins, and lignin (Liu et al., 2016). During fruit development, lignin deposition directly determines the thickness of the nutshell (Xiao et al., 2020). Traditionally, lignin is polymerized from three monomers: *p*-coumaryl alcohol, coniferyl alcohol, and sinapyl alcohol (Del Río et al., 2017). Nutshell thickness is a key trait affecting the processing quality of walnuts, as a thinner shell improves the efficiency of kernel extraction. In this study, thin-shelled elite strains such as BT-1 provide valuable parental resources for breeding thin-shelled processing varieties. Spatiotemporal analysis of lignin deposition in walnuts via microsectioning revealed that the critical lignification period occurs between 30 and 45 days after flowering (Wu X. T. et al., 2021). Future research should focus on the regulatory mechanisms of lignin synthesis, which is essential for understanding walnut shell formation and quality improvement.

### 4.3 Practical value and prospects of the BBCH phenological coding system

The BBCH phenological coding system established in this study provides substantial practical guidance for walnut production in the arid regions of Xinjiang. Notably, BT-2 demonstrates the earliest bud development and is categorized as an early-flowering variety. This variety not only flowers and sets fruit early but is also well-adapted for cultivation in areas with a reduced risk of frost (Sakar et al., 2023). Delayed flowering is acknowledged as an effective strategy to mitigate the risk of late spring frost (Sapkota et al., 2021). In contrast, BT-4 exhibits the latest leaf expansion and new shoot development, which can effectively reduce damage from late frost. In terms of irrigation management, distinct walnut varieties display significant differences in their drought response strategies, including stomatal and osmotic regulation. For certain varieties, only minimal changes in fruit quality occur under limited irrigation conditions, suggesting the potential for ‘water conservation without quality reduction’ (Xu et al., 2024).The BBCH phenological coding system employed in this study can be integrated with the fine irrigation model for practical applications. During the fruit development and hard kernel stages, the irrigation volume can be dynamically adjusted in accordance with the phenological process. Furthermore, water should be regulated during the maturation period to promote the lignification of the fruit shell and enhance the accumulation of kernel flavor (He et al., 2024).

This study presents several limitations. The phenological observation data are based on only two growing seasons. The interannual stability of traits necessitates validation through multi-year observations, particularly for quality traits such as nutshell thickness and kernel percentage, which are influenced by the interplay of multiple genes and environmental factors. Future research will aim to clarify the mechanisms by which varying temperature conditions affect the differentiation of male and female flowers as well as the fruit development of walnuts. By integrating multi-dimensional data, including photosynthetic physiological parameters, water use efficiency, and transcriptomics, this research seeks to elucidate the interaction network between early fruiting traits and quality traits. This approach will establish a more systematic theoretical foundation for the precise cultivation and molecular breeding of walnuts in the arid regions of Xinjiang.

## 5 Conclusions

Based on the extended BBCH scale, this study systematically established a standardized phenological description system for precocious walnuts in Xinjiang, covering eight principal growth stages from bud development to dormancy and 38 secondary stages. Significant differences in phenological processes were identified among different varieties and elite strains. BT-2 had the earliest budburst and the highest number of female flowers, making it suitable as an early-flowering and high-yield material. XW-185 was rich in male flower resources and suitable as a pollen donor for cross-breeding. BT-1 exhibited the highest kernel percentage and the thinnest nutshell, showing potential for the development of processing-oriented varieties. Correlation analysis of traits revealed that the trait correlation patterns of BT-2 and BT-3 were relatively complex, with opposite trends in their association directions: the number of female flowers was significantly positively correlated with the number of male inflorescences in BT-2, but significantly negatively correlated in BT-3. In contrast, XW-185 and XX-2 showed relatively independent traits. These findings provide a theoretical basis for targeted cultivation and variety improvement. The PCA and clustering heatmap results show that the six precocious walnut varieties and elite strains are clearly differentiated in key traits and classified into four clusters, offering a basis for parental selection. The established BBCH coding system can systematically guide orchard pollinizer arrangement, water and fertilizer management, and cross-breeding programs, while providing an operable technical basis for adaptability evaluation and standardized production of precocious walnuts in this region.

## Data Availability

The original contributions presented in the study are included in the article/supplementary material. Further inquiries can be directed to the corresponding authors.
